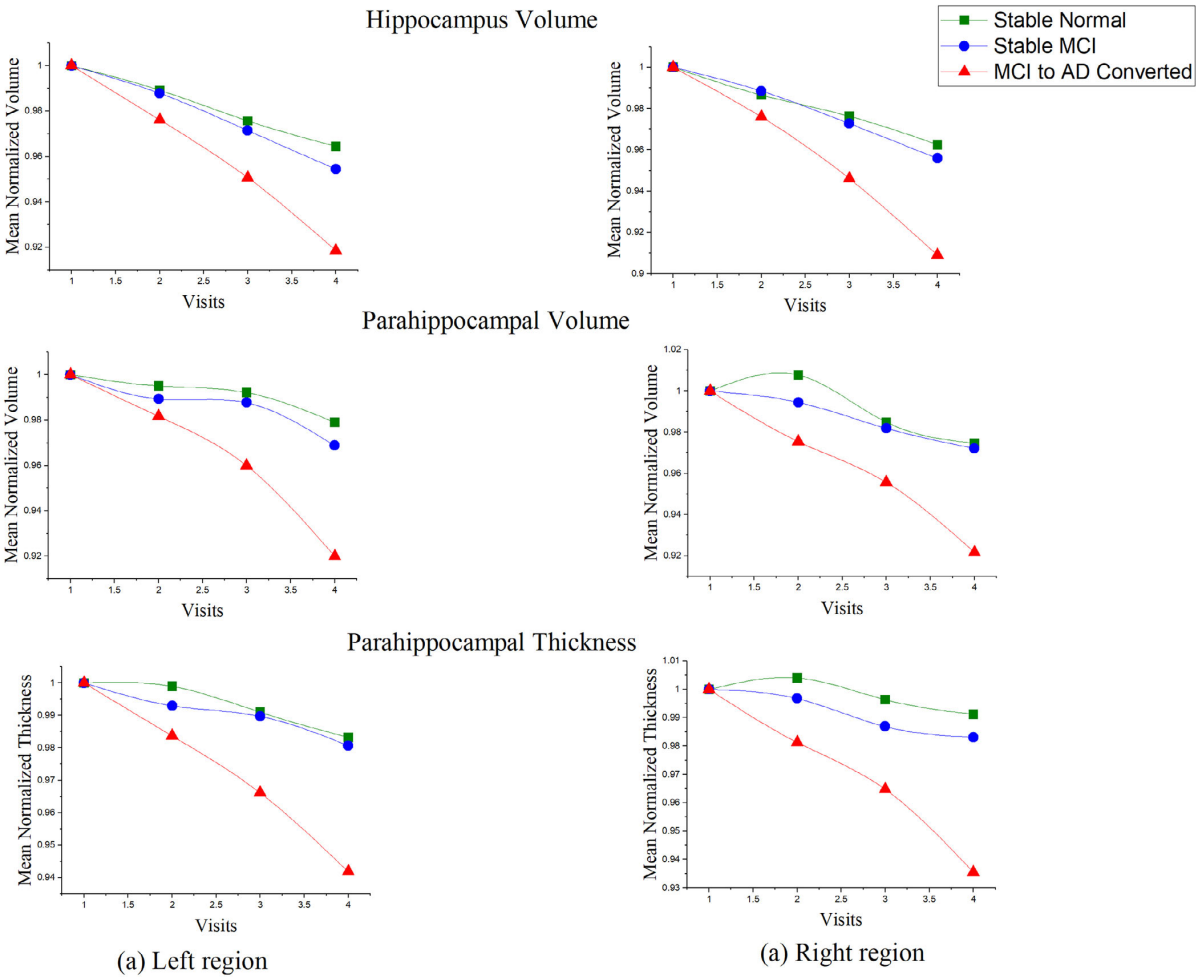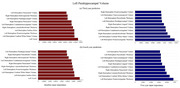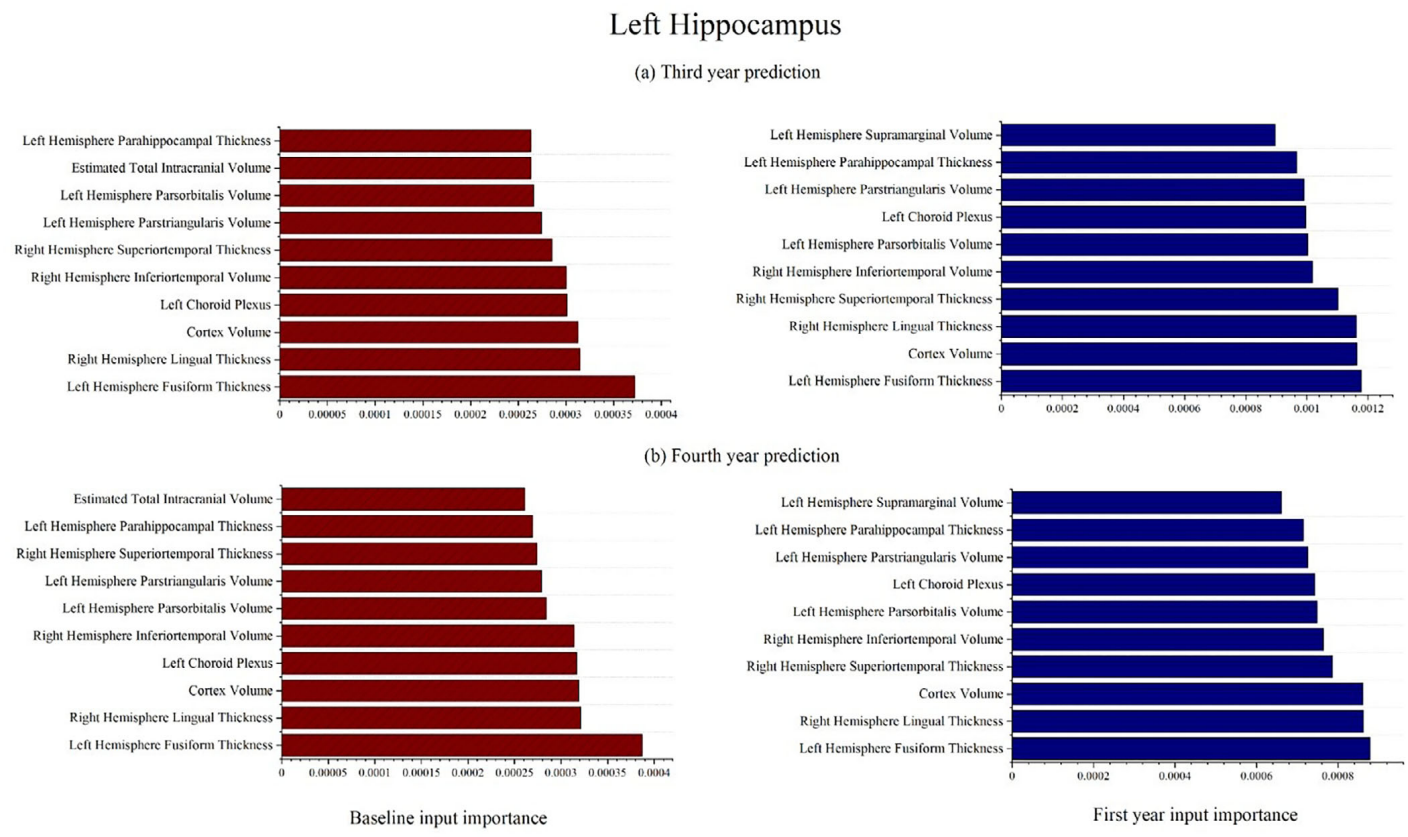# Prediction of Longitudinal Changes in Hippocampal and Parahippocampal Structures: An Interpretable Bayesian Analysis in Alzheimer's Disease

**DOI:** 10.1002/alz70856_106410

**Published:** 2026-01-09

**Authors:** Ratnadeep Das, Atri Chatterjee, Sitikantha Roy

**Affiliations:** ^1^ Indian Institute of Technology Delhi, Delhi, Delhi, India; ^2^ Vardhman Mahavir Medical College & Safdarjung Hospital, New Delhi, Delhi, India

## Abstract

**Background:**

Alzheimer's disease is characterized by progressive atrophy of hippocampus and parahippocampal structures including entorhinal cortex and parahippocampal gyrus. Predicting longitudinal volume changes remains challenging as they can occur due to normal ageing and disease progression.

**Method:**

From the ADNI dataset (*n* = 480), we used two years of baseline data including demographics, cognitive assessment scores (CDR‐SB, MMSE), and FreeSurfer‐extracted MRI measurements to predict third and fourth‐year Hippocampus volume, parahippocampal volume and thickness. We employed a GRU‐based Bayesian Encoder‐Decoder architecture (GRU‐BEND) with a 70:10:20 train‐validation‐test split. We evaluated our model over five‐fold cross‐validation and used the Integrated Gradient method for feature importance analysis. Figure 1 shows the degradation of the region's volumes over the year for different diagnosis cohorts.

**Result:**

The model demonstrated strong predictive performance for all the target variables. We employed the metrics Mean Absolute Error (MAE) and Mean Absolute Percentage Error (MAPE) to evaluate our model. As expected, the predictions for the third year were more accurate than the fourth year. MAPE for the third year ranged between 4.3% to 4.9%, while 5.1% to 6.6% for the fourth year. MAE ranged between 0.03 to 0.06. The Left and right hippocampus volume prediction was most accurate over both years (Y3: MAE: 0.039±0.002, MAPE: 4.4±0.1%; Y4: MAE: 0.045±0.003, MAPE: 5.1%). The important features contributing to the predictions for hippocampus (left) and parahippocampal volume (left) are shown below in Figure 2 and 3. The role of CDR‐SB and MMSE in the prediction was minimal. Surprisingly, amygdala and ventricle volume were among the least contributing factors for hippocampus predictions.

**Conclusion:**

In this study, we demonstrate that a GRU‐BEND model which is able to effectively predict longitudinal changes in volumes of hippocampal and parahippocampal structures in Alzheimer's disease based on two years of data. This may be helpful in prognosticating patients and quantitative estimation of treatment effects.